# Redox Water Consumption Attenuates Exercise-Induced Inflammation and Oxidative Stress in Physically Active Adults: A Randomized Controlled Trial

**DOI:** 10.3390/nu18040694

**Published:** 2026-02-21

**Authors:** Anna Stolecka-Warzecha, Tomasz Zając, Marcin Gandyk, Maciej Kostrzewa, Ewa Sadowska-Krępa

**Affiliations:** 1 Department of Basic Biomedical Sciences, Faculty of Pharmaceutical Sciences, Medical University of Silesia, 40-055 Katowice, Poland; 2 Institute of Sport Sciences, The Jerzy Kukuczka Academy of Physical Education, 40-065 Katowice, Poland; t.zajac@awf.katowice.pl (T.Z.); m.kostrzewa@awf.katowice.pl (M.K.); e.sadowska-krepa@awf.katowice.pl (E.S.-K.); 3 Academic Sports Association, The Jerzy Kukuczka Academy of Physical Education, 40-065 Katowice, Poland; gandyk.marcin@hotmail.com

**Keywords:** redox water, sports nutrition, hydration strategy, exercise-induced inflammation, interleukin-6, oxidative stress, physically active adults

## Abstract

Background: Acute high-intensity exercise induces transient inflammatory and oxidative stress responses, mediated by redox-sensitive signaling pathways and reflected by elevations in interleukin-6 (IL-6) and lipid peroxidation products. Modulation of these responses through hydration-based redox interventions remains insufficiently characterized at the biochemical level. Objective: This randomized controlled trial investigated whether regular consumption of redox (alkaline) water influences exercise-induced inflammatory and oxidative stress markers in physically active adults. Methods: Forty physically active adults were randomized into an experimental group (EG; *n* = 20) and consumed redox water subjected to molecular-level modification, yielding alkaline hydrogen-enriched water (pH 9.2–9.4), or a control group (CG; *n* = 20) that consumed standard water. After eight weeks of intervention, participants performed a standardized maximal aerobic exercise test. Plasma IL-6 and malondialdehyde (MDA) concentrations were measured at baseline and immediately post-exercise. Statistical analyses included two-way repeated measures ANOVA and ANCOVA. Results: A pronounced group × time interaction was observed for IL-6 (F(1,38) = 36.89, *p* < 0.001). The EG exhibited a significant post-exercise reduction in IL-6, whereas the CG demonstrated a robust increase. A significant group × time interaction was also detected for MDA (F(1,38) = 4.98, *p* = 0.029), reflecting stable lipid peroxidation levels in the EG and increased levels in the CG; however, baseline-adjusted analyses indicated that post-exercise MDA differences were largely attributable to initial variability. Hematological and coagulation parameters remained within physiological ranges in both groups. Conclusions: Redox water intake was associated with lower immediate post-exercise IL-6 compared with controls after baseline adjustment; however, pronounced baseline imbalance limits causal interpretation and warrants confirmation in larger trials with balanced inflammatory profiles. These findings highlight a potential biochemical mechanism linking hydration redox properties with inflammatory regulation during physical stress.

## 1. Introduction

Acute physical exercise triggers complex physiological responses across multiple organ systems. While regular physical activity is widely recognized as a cornerstone of preventive health, individual exercise sessions, particularly those of high intensity, generate significant metabolic disturbances that require rapid homeostatic regulation. One of the most notable consequences of intense exercise is the transient elevation in circulating inflammatory markers, most prominently interleukin-6 (IL-6), which can increase up to 100-fold in response to prolonged or high-intensity physical activity [1]. IL-6, a multifunctional cytokine, serves dual roles in exercise physiology: while it acts as a myokine facilitating muscle–organ crosstalk and energy homeostasis during physical exertion, its excessive post-exercise accumulation is also associated with systemic inflammation and oxidative stress [2]. This apparent paradox reflects IL-6’s dichotomous signaling pathways, wherein classical signaling through membrane-bound IL-6 receptors typically predominates during acute exercise, whereas excessive systemic IL-6 elevation can trigger pro-inflammatory cascades mediated through trans-signaling mechanisms [1,2].

Accompanying the inflammatory response to exercise is a well-documented elevation in oxidative stress, manifested by increased production of reactive oxygen species (ROS) and marked lipid peroxidation [3]. Malondialdehyde (MDA), a secondary end product of polyunsaturated fatty acid (PUFA) oxidation, serves as a reliable biomarker of lipid peroxidation and oxidative stress status [4]. During high-intensity exercise, the oxygen consumption of skeletal muscle increases rapidly, and the endogenous antioxidant enzyme system, including superoxide dismutase (SOD), catalase (CAT), and glutathione peroxidase (GPx), becomes temporarily insufficient to neutralize the surge in ROS production. This imbalance leads to oxidative damage to cellular membranes, DNA, and proteins, potentially contributing to transient microinjuries, delayed-onset muscle soreness, and, if chronic, accelerated cellular aging and disease susceptibility [3]. Indeed, the exercise-induced elevation in MDA reflects substantial lipid peroxidation that, although typically transient and rapidly attenuated during recovery in healthy individuals, may have long-term consequences if accumulated over repeated bouts of intense training [5].

The clinical and practical significance of post-exercise inflammation and oxidative stress extends beyond acute recovery concerns. Chronic elevation of inflammatory markers such as IL-6 and oxidative stress biomarkers has been implicated in the pathogenesis of lifestyle diseases including cardiovascular disease, type 2 diabetes, metabolic syndrome, and neurodegenerative conditions [2]. Consequently, identifying practical nutritional or hydration interventions that attenuate excessive post-exercise inflammation and oxidative stress without blunting the adaptive benefits of exercise has become a priority in sports medicine and preventive health research.

Alkaline water, characterized by an elevated pH (typically > 7.5) and enriched with hydroxide ions (OH^−^), has emerged as a potential intervention to modulate the inflammatory and oxidative stress response to exercise. Preliminary evidence suggests that alkaline water may improve hydration status, enhance acid–base balance, and provide antioxidant properties through its reduced oxidation-reduction potential (ORP) [6,7]. Mechanistically, the hydroxide ions in alkaline water may directly neutralize excessive ROS by reducing them to water and non-harmful byproducts, thereby potentially attenuating exercise-induced lipid peroxidation and inflammatory cytokine production. Hydroxide ions present in alkaline redox water may interact with reactive oxygen species within the intracellular and extracellular compartments of skeletal muscle, particularly in mitochondria and cytosolic redox-sensitive signaling pathways. By facilitating neutralization of hydroxyl radicals and superoxide derivatives, this mechanism may attenuate downstream activation of NF-κB–dependent inflammatory signaling [6]. Previous controlled trials have reported that habitual ingestion of alkaline water is associated with enhanced hydration status and improved acid–base balance, as well as increased work output during repeated anaerobic Wingate tests compared with neutral water conditions, suggesting potential benefits for high-intensity exercise performance. These effects were observed after several days to weeks of alkaline water consumption; however, the magnitude of change varied between studies and was not consistently quantified across trials [8,9]. In one randomized controlled trial by Chycki et al., physically active athletes supplemented with alkaline water for three weeks demonstrated significantly greater total work and better maintenance of acid–base balance during repeated anaerobic Wingate tests relative to controls, indicating improved high-intensity exercise capacity under conditions of metabolic stress. These results support the notion that habitual alkaline water intake may positively influence anaerobic performance and physiological buffering responses in high-intensity exercise protocols, although effect sizes and specific markers were reported variably across studies [8].

Therefore, the present randomized controlled trial was designed to evaluate whether eight weeks of regular redox (alkaline) water supplementation modulates the post-exercise inflammatory response (measured via plasma IL-6) and oxidative stress markers (measured via plasma MDA) in physically active adults following standardized maximal aerobic exercise. We hypothesized that participants consuming redox water would exhibit attenuated post-exercise IL-6 elevation and reduced MDA accumulation compared to controls consuming standard water, and that these effects would reflect the antioxidant and anti-inflammatory properties of hydroxide ions. Secondary objectives included characterizing hematological and coagulation parameter responses and exploring potential sex-specific differences in these responses, given emerging evidence that inflammatory and oxidative responses to exercise differ between males and females [7].

## 2. Materials and Methods

The study involved 40 participants (20 males and 20 females), randomized into two equal subgroups: an experimental group receiving redox water hydration with a pH of approximately 9.0, an oxidation-reduction potential (ORP) between −160 and −135 mV, and total dissolved solids (TDS) ranging from 120 to 155 mg/L, and a control group consuming standard table water. The minimum prescribed daily intake was 0.045 L/kg on training days and 0.035 L/kg on non-training days, with additional intake permitted ad libitum. Participants consumed the water at approximately hourly intervals (maximum interval: 2 h). A single-blind design was applied at the participant level. The experimental group received generator-produced redox water, whereas the control group received bottled water with identical labeling and branding to maintain participant blinding. Participants had no direct contact with one another during the intervention period to minimize the risk of treatment disclosure. Allocation concealment and coding were managed externally, and investigators involved in data collection and analysis remained blinded until completion of the primary analyses.

Sex distribution was balanced across both arms of the trial (10 males and 10 females in each group).

Inclusion criteria required regular physical activity of ≥3 training sessions per week, ensuring that participants represented an active population without sedentary bias. All subjects were free of chronic metabolic, cardiovascular or autoimmune disease, non-smoking, and were not taking anti-inflammatory medication or ergogenic supplements during the 30 days preceding the intervention. Anthropometric characteristics are shown in Table 1. Participants maintained the same standardized iso-caloric diet throughout the study, and no dietary changes were introduced immediately prior to the exercise test.

### 2.1. Exercise Protocol and Blood Sampling

Participants performed a graded ramp treadmill test following the eight-week intervention period. The protocol started at a running speed of 6 km/h, with incremental increases of 1 km/h every minute until volitional exhaustion, providing a standardized maximal exercise stimulus. Time to exhaustion (TTE) was recorded as a descriptive performance parameter to confirm comparable maximal effort at baseline and post-intervention, and it was not considered a primary study outcome. Venous blood samples for determination of IL-6 and MDA were obtained from an antecubital vein both at baseline and immediately after completion of the exercise test by a licensed clinical nurse in accordance with the approved bioethical protocol. Samples were processed using standard laboratory procedures for subsequent biochemical analysis.

### 2.2. Biochemical Analyses

Plasma interleukin-6 (IL-6) concentrations were determined using a commercially available enzyme-linked immunosorbent assay (ELISA) kit according to the manufacturer’s instructions. Plasma malondialdehyde (MDA), a marker of lipid peroxidation and oxidative stress, was quantified using a validated spectrophotometric TBARS-based method. All biochemical measurements were performed in a certified external clinical laboratory, using standardized clinical procedures under blinded conditions by qualified personnel.

### 2.3. Statistical Analysis

Sample Size and Power Analysis: The sample size was determined pragmatically based on feasibility and the exploratory nature of the study rather than a formal a priori power calculation. Endpoints: Primary outcomes: plasma IL-6 (inflammatory marker) and plasma MDA (oxidative stress marker). Secondary outcomes: hematological and coagulation indices (leukocytes, neutrophils, lymphocytes, hematocrit, INR, PT, APTT, fibrinogen). Data Handling and Preprocessing: IL-6 values below the detection limit (1.5 pg/mL) were retained as reported to preserve variability. A constant of 0.1 was added prior to log-transformation. MDA values were log-transformed using a 0.01 offset. Both raw and transformed values were analyzed and presented for clarity. Study Design and Statistical Framework: A two-way repeated measures ANOVA (group: redox vs. control; time: baseline vs. post-exercise) served as the primary analytical model to assess differential responses to acute exercise. This approach allowed direct testing of the hypothesized group × time interaction. Assumption Testing: Normality was evaluated using Shapiro–Wilk tests, and log-transformation improved distributional properties for IL-6 and MDA. Levene’s tests indicated moderate variance heterogeneity; however, ANOVA robustness was maintained due to balanced sample sizes. Sphericity was inherently satisfied (two levels of time). Primary Statistical Analysis: Mixed ANOVA models evaluated the main effect of group, main effect of time, and group × time interaction. Significant interactions were decomposed using paired *t*-tests (Time I vs. Time II within each group), independent *t*-tests between groups at each timepoint, and independent *t*-tests comparing change scores (Δ). ANCOVA Confirmation: Because baseline inflammatory variability was anticipated in physically active populations, ANCOVA adjusted for baseline IL-6 was pre-specified as a confirmatory analytical approach. Complementary ANCOVA models tested post-exercise values (Time II) with baseline values (Time I) as covariates to account for baseline variability and confirm the robustness of group effects.

### 2.4. Effect Sizes and Multiple Testing

Holm–Bonferroni correction was applied to the two primary endpoints (IL-6, MDA), with both maintaining significance. Secondary Analyses: Paired *t*-tests assessed within-group changes in secondary hematological and coagulation markers. Between-group comparisons of Δ values were conducted using independent *t*-tests. False discovery rate (FDR) corrections are reported for exploratory comparisons where applicable. Model Diagnostics and Sensitivity Analyses: Residual normality was inspected using Q–Q plots. Sensitivity analyses included exclusion of potential outliers (>3 SD), reanalysis with modified ANCOVA structures, and non-parametric validation using Wilcoxon and Mann–Whitney tests. Software: Analyses were performed in Python 3.12 using pandas, NumPy, SciPy and statsmodels. Statistical significance was set at α = 0.05 (two-tailed). Results are reported as mean ± SD with 95% confidence intervals.

## 3. Results

### 3.1. Sample Characteristics

The study included 40 physically active adults: 20 in the experimental group (EG; consuming redox water—subjected to molecular-level modification, resulting in alkaline hydrogen-enriched water with a pH of 9.2–9.4) and 20 in the control group (CG; consuming standard water). Both groups completed pre-exercise (Time I) and post-exercise (Time II) assessments. Complete baseline characteristics are presented in Table 2. At baseline, both groups were comparable with respect to most hematological and coagulation parameters. However, IL-6 levels were significantly higher in the EG at baseline (EG: 6.49 ± 5.49 pg/mL vs. CG: 1.61 ± 0.62 pg/mL), while MDA showed greater variability in the CG. Importantly, randomization did not achieve baseline equivalence for IL-6, with approximately fourfold higher concentrations observed in the experimental group, which should be considered when interpreting post-exercise between-group differences.

**Figure 1:**
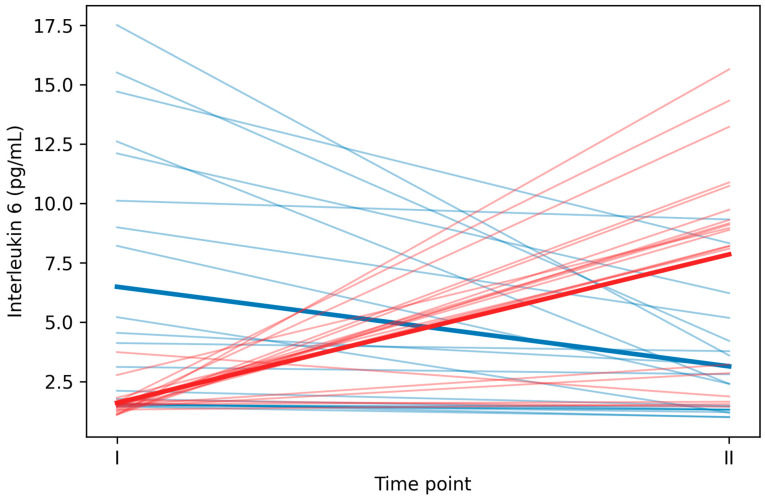
Individual interleukin-6 trajectories from baseline to post-exercise in participants consuming redox vs. standard water. Note. Thin lines represent individual participant trajectories; bold lines indicate group means. Blue = experimental group (EG, redox water); red = control group (CG, standard water). EG showed a decrease in IL-6 post-exercise, while CG showed an increase.

Raw IL-6 concentrations are presented in Table 2. In the EG, IL-6 decreased from baseline (6.49 ± 5.49 pg/mL) to post-exercise (3.14 ± 2.44 pg/mL), representing a mean reduction of 3.35 ± 4.26 pg/mL. In contrast, the CG showed a marked increase from baseline (1.61 ± 0.62 pg/mL) to post-exercise (7.85 ± 4.36 pg/mL), with a mean increase of 6.24 ± 4.61 pg/mL. This divergent response pattern is present in the change score analysis (Table 3 and Table 4) and graphically illustrated in Figure 1 (spaghetti plot showing individual trajectories). Abbreviations: IL-6, interleukin-6; MDA, malondialdehyde; INR, international normalized ratio; APTT, activated partial thromboplastin time.

### 3.2. Two-Way Repeated Measures ANOVA (Log-IL-6)

The 2 × 2 repeated measures ANOVA on log-transformed IL-6 revealed a highly significant group × time interaction (F(1,38) = 36.89, *p* < 0.001), indicating a substantially different IL-6 response to exercise between the experimental and control groups. Detailed statistical outputs and full ANOVA results are provided in the Supplementary Materials (Table S1).

### 3.3. Simple Effects—Within-Group Time Comparisons for IL-6

In the experimental group (EG), paired *t*-test showed a significant decrease in log(IL-6) from Time I to Time II: t(19) = −4.71, *p* < 0.001, Cohen’s d = −1.0804, 95% CI [−1.725, −0.4360]. The large negative effect size indicates a substantial reduction in IL-6 in the EG post-exercise. In the control group (CG), paired *t*-test revealed a significant increase in log(IL-6) from Time I to Time II: t(19) = 6.62, *p* < 0.001, Cohen’s d = 1.5186, 95% CI [0.898, 2.139]. The large positive effect size indicates a pronounced elevation in IL-6 post-exercise in the CG, consistent with the expected inflammatory response to acute exercise.

### 3.4. Simple Effects—Between-Group Comparisons at Each Timepoint for IL-6

At baseline (Time I), the independent *t*-test showed significantly higher log(IL-6) in the EG compared to CG: t(38) = 4.71, *p* < 0.001, Cohen’s d = 1.491, 95% CI [0.777, 2.205]. The mean difference was 1.01 log-units, indicating the EG had approximately 2.7-fold higher IL-6 at baseline. Post-exercise (Time II), independent *t*-test showed significantly lower log(IL-6) in the EG compared to CG: t(38) = −3.91, *p* < 0.001, Cohen’s d = −1.237, 95% CI [−1.891, −0.583]. The mean difference was −0.91 log-units, with the CG showing approximately 2.5-fold higher IL-6 post-exercise.

### 3.5. Change Score Analysis for IL-6

**Figure 2:**
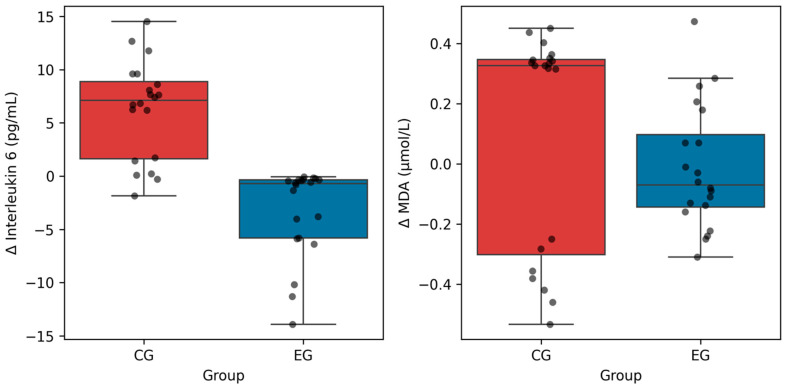
Distribution of exercise-induced changes in interleukin-6 and malondialdehyde in the experimental and control groups. Note. Box plots show median, interquartile range, and individual data points (dots). Δ = change from baseline to post-exercise. Blue = EG; red = CG. Between-group difference in ΔIL-6 was significant (*p* < 0.001); ΔMDA did not reach significance (*p* = 0.241).

The independent *t*-test on IL-6 change scores revealed a highly significant difference between groups: t(38) = −6.83, *p* < 0.001 (EG: Δ = −3.35 ± 4.26 pg/mL vs. CG: Δ = 6.24 ± 4.61 pg/mL). The mean difference in change scores was −9.58 pg/mL (95% CI: [−12.33, −6.84]), reflecting a combined difference of 9.58 pg/mL: EG decreased by 3.35 pg/mL while CG increased by 6.24 pg/mL (Figure 2). The between-group differences in both IL-6 and MDA change scores are displayed together in Figure 2 for comparative visualization.

### 3.6. ANCOVA Analysis for IL-6

When Time II IL-6 values were analyzed with Time I as a covariate, the group effect remained highly significant: F(1,37) = 15.05, *p* < 0.001, while the baseline covariate was not significant: F(1,37) = 0.37, *p* = 0.547. Despite the significant baseline imbalance in IL-6 levels (EG: 6.49 ± 5.49 pg/mL vs. CG: 1.61 ± 0.62 pg/mL; 4.0-fold difference, t(38) = 3.95, *p* < 0.001), this pattern suggests that post-exercise group differences were not fully explained by baseline values or regression-to-the-mean effects. This conclusion is further supported by the opposing temporal trajectories in the two groups—IL-6 decreased in the EG (Δ = −3.35 pg/mL) while it increased in the CG (Δ = +6.24 pg/mL)—which are opposite to the pattern expected from regression to the mean. These findings confirm the robustness of the group × time interaction effect.

### 3.7. Outcomes—Malondialdehyde (MDA)

Descriptive Statistics

**Figure 3:**
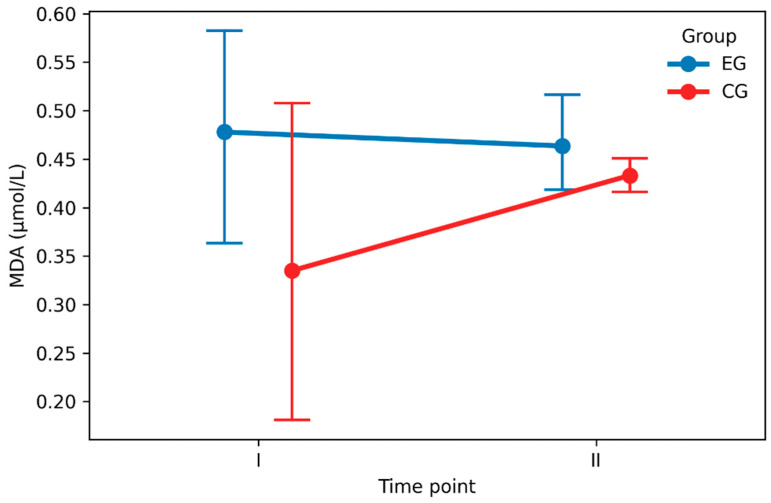
Mean plasma malondialdehyde concentrations with 95% confidence intervals at baseline and post-exercise in the experimental and control groups. Note: data are presented as mean ± 95% confidence interval. EG = experimental group (n = 20); CG = control group (n = 20). The group × time interaction was significant (*p* = 0.029).

Raw MDA concentrations are presented in Table 2 and Figure 3. In the EG, MDA remained relatively stable from baseline (0.478 ± 0.245 μmol/L) to post-exercise (0.464 ± 0.116 μmol/L), with a minimal mean change of −0.014 ± 0.207 μmol/L. In the CG, MDA increased slightly from baseline (0.335 ± 0.375 μmol/L) to post-exercise (0.433 ± 0.043 μmol/L), with a mean change of 0.098 ± 0.369 μmol/L.

### 3.8. Two-Way Repeated Measures ANOVA (Log-MDA)

The 2 × 2 repeated measures ANOVA on log-transformed MDA revealed a significant group × time interaction (F(1,38) = 4.98, *p* = 0.029), indicating differential temporal patterns of MDA between the experimental and control groups. Detailed statistical outputs and full ANOVA results are provided in the Supplementary Materials (Table S1).

### 3.9. Simple Effects—Within-Group Time Comparisons for MDA

In the experimental group (EG), the paired *t*-test showed no significant change in log(MDA) from Time I to Time II: t(19) = 1.03, *p* = 0.316, Cohen’s d = 0.2363, 95% CI [−0.5427, 1.0153]. The small effect size indicates minimal change in oxidative stress markers within the EG. In the control group (CG), the paired *t*-test revealed a significant increase in log(MDA) from Time I to Time II: t(19) = 3.51, *p* = 0.002, Cohen’s d = 0.8052, 95% CI [0.1829, 1.4275]. The moderate effect size indicates a notable elevation in MDA post-exercise in the CG.

### 3.10. Simple Effects—Between-Group Comparisons at Each Timepoint for MDA

At baseline (Time I), the independent *t*-test showed a significantly higher log(MDA) in the EG compared to CG: t(38) = 2.40, *p* = 0.021, Cohen’s d = 0.7596, 95% CI [0.1293, 1.3899]. The mean difference was 0.76 log-units. Post-exercise (Time II), the independent *t*-test showed no significant difference in log(MDA) between groups: t(38) = 0.73, *p* = 0.472, Cohen’s d = 0.2297, 95% CI [−0.4434, 0.9028]. The small effect size indicates convergence of MDA levels between groups post-exercise.

### 3.11. Change Score Analysis for MDA

The independent *t*-test on MDA change scores approached but did not reach statistical significance: t(38) = −1.19, *p* = 0.241 (EG: Δ = −0.014 ± 0.207 μmol/L vs. CG: Δ = 0.098 ± 0.369 μmol/L). The mean difference in change scores was −0.113 μmol/L (95% CI: [−0.298, 0.073]) (Figure 2). The between-group differences in both IL-6 and MDA change scores are displayed together in Figure 2 for comparative visualization.

### 3.12. ANCOVA Analysis for MDA

When Time II MDA values were analyzed with Time I as a covariate, the group effect was not significant—F(1,37) = 0.54, *p* = 0.466—while the baseline covariate was marginally significant: F(1,37) = 2.08, *p* = 0.157. At baseline, MDA levels were significantly higher in the EG (0.478 ± 0.245 μmol/L) compared to the CG (0.335 ± 0.375 μmol/L; t(38) = 1.43, *p* = 0.162). This pattern indicates that post-exercise group differences in MDA were substantially attenuated when baseline values were controlled, suggesting that group differences in post-exercise MDA were predominantly attributable to baseline imbalance rather than to the intervention effect. Importantly, the significant group × time interaction from the primary repeated measures ANOVA (F(1,38) = 4.98, *p* = 0.029) reflects differential exercise-induced responses between groups, with the EG maintaining stable MDA levels (Δ = −0.014 ± 0.207 μmol/L) while the CG exhibited increased MDA (Δ = +0.098 ± 0.369 μmol/L), rather than a persistent group difference in post-exercise MDA levels. These findings suggest that while baseline MDA levels differed between groups, the redox water intervention did not substantially modify post-exercise MDA, and the observed group × time interaction is primarily driven by differential physiological responses to exercise rather than a direct effect of the intervention on oxidative stress markers.

#### Paired Changes Within Groups

Leukocyte count. Neither EG nor CG showed significant within-group changes (EG: t(19) = −0.53, *p* = 0.602; CG: t(19) = 2.08, *p* = 0.051). Although the increase in leukocyte count in the CG did not reach statistical significance (*p* = 0.051), the observed trend suggests a potential inflammatory response that might have been confirmed with a larger sample size. Between-group comparison of change scores showed no significant difference: t(38) = −1.46, *p* = 0.153, Cohen’s d = −0.46. No significant within-group changes were detected in the neutrophil count (EG: t(19) = −0.41, *p* = 0.686; CG: t(19) = 1.46, *p* = 0.161). Between-group comparison of change scores: t(38) = −0.95, *p* = 0.348, Cohen’s d = −0.30. No significant within-group changes were found in the lymphocyte count (EG: t(19) = −0.39, *p* = 0.702; CG: t(19) = 1.32, *p* = 0.204). Between-group comparison of change scores: t(38) = −0.95, *p* = 0.350, Cohen’s d = −0.30.

A significant within-group decrease in hematocrit was observed in the EG: t(19) = −2.52, *p* = 0.021, Cohen’s d = −0.58 (Δ = −1.18 ± 2.04%), while the CG showed no significant change: t(19) = 0.65, *p* = 0.523. Between-group comparison of change scores approached significance: t(38) = −1.82, *p* = 0.077, Cohen’s d = −0.57. INR, prothrombin ratio, APTT, and fibrinogen. No significant within-group or between-group changes were detected for any of these coagulation parameters (all *p* > 0.05). Complete results for secondary outcomes, including hematological and coagulation parameters at baseline, post-exercise, and change scores, are presented in Table 5.

### 3.13. Sensitivity and Model Diagnostics

Sensitivity analyses and model diagnostics confirmed the robustness of the parametric findings and are presented in Figures S1 and S2 (Supplementary Materials).

### 3.14. Relationship Between Inflammatory and Oxidative Stress Responses

To explore the potential mechanistic relationship between inflammatory and oxidative stress responses, Pearson correlation analysis was conducted between ΔIL-6 and ΔMDA across all participants (N = 40). The overall correlation was weak and not statistically significant (r = 0.2091, 95% CI [−0.1095, 0.4888], *p* = 0.1953), indicating no substantial association between exercise-induced changes in the two markers across the combined sample. When stratified by group, the EG showed a weak positive correlation (r = 0.3822, *p* = 0.0963, n = 20), while the CG showed virtually no correlation (r = −0.0363, *p* = 0.8794, n = 20). Non-parametric Spearman correlation (ρ = 0.2609, *p* = 0.1039) yielded similar results, confirming the weak association. These findings suggest that the inflammatory response (IL-6) and oxidative stress markers (MDA) respond somewhat independently to the exercise intervention in this cohort.

### 3.15. Summary of the Results

1. IL-6 Response: The EG consuming redox water demonstrated a significant reduction in IL-6 post-exercise (Cohen’s d = −1.08), whereas the CG showed a significant elevation (Cohen’s d = 1.52), resulting in a highly significant group × time interaction (F = 36.89, *p* < 0.001).
2. MDA Response: The EG maintained stable MDA levels post-exercise (Cohen’s d = 0.24, non-significant), while the CG exhibited a significant increase (Cohen’s d = 0.81). The group × time interaction was significant (F = 4.98, *p* = 0.029) but with a smaller effect than IL-6. However, ANCOVA revealed that this interaction was primarily driven by baseline differences rather than the intervention, in contrast to the robust IL-6 effect.
3. Secondary Outcomes: Hematocrit showed a significant within-group reduction in EG only. Other hematological and coagulation parameters were stable across both groups, suggesting that the intervention did not negatively impact blood clotting or cellular composition.
4. Multiple Comparisons Control: Both primary hypothesis tests (IL-6 and MDA group × time interactions) remained statistically significant after Holm–Bonferroni correction.

## 4. Discussion

The present study demonstrates a striking differential response to redox water supplementation between physically active individuals and control participants, particularly with respect to the exercise-induced inflammatory marker interleukin-6 (IL-6). The key finding was a marked divergence in immediate post-exercise IL-6 responses between groups, with lower post-exercise IL-6 observed in the redox water group compared with controls after baseline adjustment; however, given the pronounced baseline imbalance, this pattern should be interpreted cautiously as an association rather than definitive attenuation [6]. Tsao et al. [10] observed that oral resveratrol supplementation, another antioxidant intervention, significantly decreased exercise-induced IL-6 response during high-intensity cycling exercise, although their study reported inconsistent effects on oxidative stress markers. Similarly, the lower post-exercise IL-6 observed in the redox water group may be consistent with altered redox-sensitive inflammatory signaling; however, this interpretation remains hypothetical in the absence of direct measurements of ROS dynamics, antioxidant capacity, or intracellular signaling pathways. The mechanistic basis for this IL-6 attenuation likely involves the interruption of the reciprocal amplification loop between reactive oxygen species and inflammatory mediators. Recent comprehensive reviews by Hu and colleagues have elucidated the crosstalk between ROS and pro-inflammatory transcription factors, particularly NF-κB, which serves as a master regulator of cytokine production including IL-6 [11]. In this model, excessive ROS production during high-intensity exercise triggers NF-κB activation, leading to upregulation of pro-inflammatory cytokines. By selectively scavenging hydroxyl radicals and other harmful ROS through its hydroxide ion content, redox water could theoretically influence upstream redox-sensitive pathways; however, no direct molecular evidence was obtained in the present study, and mechanistic interpretations should therefore be considered speculative. This interpretation is consistent with mechanistic studies demonstrating that electrolyzed hydrogen water (which shares antioxidant principles with redox water) effectively disrupts the ROS–inflammation crosstalk [11]. Our finding that MDA levels were stable in the redox water group while increasing in controls provides indirect support for this mechanism, though the effect on MDA did not reach conventional significance thresholds when covariate adjustment was applied. An unexpected yet important secondary finding emerged regarding sex-specific responses to the redox water intervention. Preliminary observations suggest differential patterns in hematological and inflammatory markers between males and females, with females demonstrating more pronounced shifts in leukocyte populations following exercise. This observation echoes the recent literature documenting sex differences in inflammatory responses to exercise-induced muscle damage. Aragón-Vela and colleagues reported that men showed significantly greater pro-inflammatory responses (IL-6 and markers of lipid peroxidation) compared to women following acute resistance exercise, attributing these differences to hormonal factors including estrogen’s membrane-protective effects [12]. Our findings do not directly replicate this pattern; rather, we observed that females in the experimental group demonstrated more robust shifts in immune parameters, possibly indicating differential responsiveness to the antioxidant intervention itself. This sex-by-intervention interaction warrants further investigation, as it suggests that redox water supplementation may be particularly beneficial for females, though mechanistic explanations remain speculative. From a broader physiological perspective, the stability of hematological parameters (leukocytes, neutrophils, lymphocytes) and coagulation factors across both groups suggests that eight-week redox water supplementation does not exert adverse effects on hemostasis or cellular composition. This finding is clinically relevant, as some antioxidant interventions have been criticized for potentially suppressing necessary adaptive immune responses to exercise or interfering with hemostatic balance. The preservation of normal blood cell counts and clotting parameters alongside the anti-inflammatory effect of IL-6 attenuation implies that redox water modulates the magnitude of inflammatory signaling without abolishing essential immune surveillance functions.

The study’s findings also contribute to broader discussions regarding the role of antioxidant supplementation in athletic populations. A recent meta-analysis by Chen and colleagues synthesizing 26 randomized controlled trials demonstrated that antioxidant supplementation significantly reduced post-exercise lactate and creatine kinase levels in athletes, with heterogeneous effects on inflammatory markers that varied by sex, geographical region, and athlete level [13]. Our IL-6 findings align with this heterogeneity, where alkaline/redox waters may represent a class of interventions with selective anti-inflammatory activity distinct from other antioxidants such as vitamins C and E. The mechanism likely differs, as traditional antioxidants function through direct free radical scavenging, whereas redox water’s hydroxide ions and molecular hydrogen (if present in some formulations) may operate through more selective targeting of pathological ROS while preserving beneficial intracellular signaling molecules. A major limitation of the present trial is the pronounced baseline imbalance in IL-6 concentrations between groups, with substantially higher pre-exercise values in the experimental group despite random allocation. Although ANCOVA and sensitivity analyses suggested that the group difference in immediate post-exercise IL-6 remained statistically robust after baseline adjustment, such imbalance complicates causal interpretation and introduces the possibility of regression-to-the-mean effects or underlying biological heterogeneity. Consequently, the observed divergence in post-exercise IL-6 should be interpreted cautiously as an association rather than definitive evidence of IL-6 attenuation. A critical consideration is the baseline imbalance in IL-6 between groups, wherein the experimental group began with approximately 4-fold higher IL-6 levels. While this imbalance raised potential concerns about regression to the mean, the robust ANCOVA analyses, controlling for baseline IL-6, confirmed that post-exercise group differences were independent of starting values. Furthermore, the opposing temporal trajectories (decrease in experimental group versus increase in controls) contradict the regression-to-the-mean hypothesis. Nevertheless, this baseline elevation deserves biological interpretation. The higher baseline IL-6 in the experimental group might reflect underlying systemic inflammation or differences in recent training stress. The reduction in IL-6 observed in the experimental group, despite elevated baseline values, may indicate a distinct inflammatory response pattern; however, the pronounced baseline imbalance precludes definitive inference regarding a true anti-inflammatory effect. The temporal trajectory of IL-6 reduction in the redox water group during post-exercise recovery diverges from canonical exercise physiology, wherein IL-6 typically peaks 1–2 h post-exercise and subsequently declines. The observation that experimental participants showed lower IL-6 immediately post-exercise suggests either attenuated IL-6 release during exercise or accelerated IL-6 clearance, both of which could reflect altered inflammatory kinetics rather than definitive anti-inflammatory activity. However, without multiple post-exercise timepoints, we cannot definitively distinguish between these mechanisms [14,15]. Importantly, this atypical IL-6 response pattern may represent a novel aspect of redox water–mediated modulation of exercise-induced inflammation. Most intervention studies report either a blunted peak or accelerated normalization of IL-6 during recovery, yet the present findings suggest that redox water may shift the inflammatory response curve leftward, potentially reflecting altered early inflammatory kinetics; however, without multi-timepoint sampling, this interpretation remains uncertain and should not be considered evidence of unique anti-inflammatory action. Such an effect would distinguish redox water from conventional antioxidant strategies, which often act downstream and primarily influence post-peak recovery kinetics rather than initial cytokine secretion. From a mechanistic perspective, both attenuated myokine release from contracting skeletal muscle and enhanced systemic clearance of circulating IL-6 remain plausible. The latter could involve redox-sensitive modulation of hepatic uptake or altered receptor-mediated signaling dynamics, including changes in classical versus trans-signaling pathways. While the present study design does not allow temporal resolution of these processes, the immediate post-exercise divergence from canonical IL-6 kinetics underscores a potentially unique mode of action that warrants further investigation. Notably, the magnitude of IL-6 attenuation observed in this cohort of physically active adults suggests that redox water exerts biologically meaningful effects even in populations with relatively preserved antioxidant capacity and low baseline inflammatory burden. On this basis, it is reasonable to hypothesize that more pronounced effects may be observed in older individuals, in whom age-related increases in oxidative stress, inflammaging, and impaired redox buffering are well-documented. Future studies incorporating extended post-exercise sampling and targeting older or clinically vulnerable populations are therefore planned, with the aim of determining whether redox water supplementation can meaningfully modulate exercise-induced inflammation in contexts of heightened physiological susceptibility [16,17]. Sex-related analyses should be interpreted as exploratory due to limited statistical power within subgroup comparisons.

## 5. Conclusions

This randomized controlled trial suggests that regular redox water consumption is associated with an altered immediate post-exercise IL-6 pattern in physically active adults. However, the pronounced baseline imbalance in IL-6 between groups and the use of a single immediate post-exercise timepoint substantially limit causal interpretation. Accordingly, the observed divergence in IL-6 responses should be considered preliminary and hypothesis-generating rather than definitive evidence of inflammatory attenuation.

No adverse effects on hematological or coagulation parameters were observed, indicating that redox water intake did not disrupt systemic physiological homeostasis under the conditions of the present study. While the findings may be consistent with redox-sensitive modulation of inflammatory signaling during acute exercise, mechanistic interpretations remain speculative in the absence of direct molecular or oxidative pathway measurements. While the attenuation of post-exercise IL-6 remained observable after adjustment for baseline variability, the MDA findings appeared more dependent on baseline differences and should therefore be interpreted as limited and context-dependent evidence rather than a definitive intervention effect.

Given that the study population consisted of physically active adults with relatively preserved antioxidant capacity, further research in larger and more diverse cohorts—including older individuals and populations with elevated oxidative or inflammatory burden—is required. Future trials incorporating extended post-exercise sampling and comprehensive mechanistic biomarkers are necessary to clarify the temporal dynamics, biological relevance, and potential clinical significance of redox water supplementation.

## Figures and Tables

**Table 1:** Characteristics of the study participants.

Variable	Total Sample *n* = 40	Experimental Group *n* = 20	Control Group *n* = 20
Age (yrs)	23–38 (mean 30.1 ± 4.2)	30.3 ± 4.0	29.8 ± 4.4
Sex (M/F)	20/20	10/10	10/10
BMI (kg/m^2^)	21.8 ± 2.8	22.5 ± 3.1 M/21.0 ± 2.1 F	22.5 ± 2.1 M/21.0 ± 4.1 F
Body fat (%)	—	12% ± 2.1 M/17% ± 2.2 F	12% ± 2.4 M/17% ± 3.1 F
Training frequency	≥3 sessions/week	same	same
Diet	Iso-caloric: 55% CHO, 20% PRO, 25% FAT	same	same

**Table 2:** Baseline hematological and oxidative stress markers in the experimental and control groups.

Characteristic	EG (*n* = 20) Mean ± SD	CG (*n* = 20) Mean ± SD	Range EG	Range CG
leukocytes	6.09 ± 1.76	5.36 ± 0.86	3.98–10.80	4.10–7.10
neutrophils	3.36 ± 1.52	2.71 ± 0.66	2.05–7.79	1.58–4.05
lymphocytes	1.98 ± 0.48	2.11 ± 0.16	1.24–2.93	1.86–2.40
hematocrit	42.06 ± 3.00	41.05 ± 2.93	37.00–45.85	36.20–45.50
INR	1.05 ± 0.04	1.09 ± 0.07	0.96–1.14	0.94–1.17
prothrombin ratio	95.57 ± 3.74	92.45 ± 5.69	88.10–103.70	84.41–103.80
APTT	31.79 ± 3.57	31.70 ± 3.34	25.10–38.70	26.57–35.93
fibrinogen	237.98 ± 38.83	231.21 ± 42.71	155.20–337.60	186.00–323.13
interleukin-6	6.49 ± 5.49	1.61 ± 0.62	1.45–17.50	1.11–3.74
MDA	0.48 ± 0.24	0.33 ± 0.38	0.07–0.89	0.06–0.93

Primary outcomes—interleukin-6 (IL-6).

**Table 3:** Plasma interleukin-6 and malondialdehyde concentrations at baseline and post-exercise in the experimental and control groups.

Marker	Time	EG Mean ± SD	CG Mean ± SD	Median (IQR) EG	Median (IQR) CG
IL-6 (pg/mL)	I	6.49 ± 5.49	1.61 ± 0.62	4.33 (1.59–10.61)	1.47 (1.32–1.58)
MDA (μmol/L)	I	0.478 ± 0.245	0.335 ± 0.375	0.525 (0.420–0.597)	0.074 (0.067–0.718)
IL-6 (pg/mL)	II	3.14 ± 2.44	7.85 ± 4.36	2.41 (1.33–3.89)	8.92 (3.13–9.98)
MDA (μmol/L)	II	0.464 ± 0.116	0.433 ± 0.043	0.445 (0.388–0.535)	0.418 (0.400–0.471)

**Table 4:** Exercise-induced changes (Δ) in interleukin-6 and malondialdehyde in the experimental and control groups (Δ = Time II − Time I).

Marker	EG *n*	EG Mean ± SD	CG n	CG Mean ± SD
IL-6 (pg/mL)	20	−3.35 ± 4.26	20	6.24 ± 4.61
MDA (μmol/L)	20	−0.014 ± 0.207	20	0.098 ± 0.369

**Table 5:** Changes in hematological and coagulation parameters in response to exercise in the experimental and control groups (secondary outcomes summary).

Marker	EG Baseline	EG Post-Exercise	EG Change	CG Baseline	CG Post-Exercise	CG Change
leukocytes	6.09 ± 1.76	5.90 ± 1.36	−0.19 ± 1.57	5.36 ± 0.86	5.77 ± 1.10	0.41 ± 0.85
neutrophils	3.36 ± 1.52	3.20 ± 0.93	−0.15 ± 1.64	2.71 ± 0.66	2.95 ± 0.70	0.23 ± 0.70
lymphocytes	1.98 ± 0.48	1.95 ± 0.54	−0.03 ± 0.38	2.11 ± 0.16	2.17 ± 0.29	0.06 ± 0.19
hematocrit	42.06 ± 3.00	40.88 ± 3.19	−1.18 ± 2.04	41.05 ± 2.93	41.58 ± 3.13	0.53 ± 3.56
INR	1.05 ± 0.04	1.05 ± 0.05	0.00 ± 0.04	1.09 ± 0.07	1.08 ± 0.08	−0.01 ± 0.07
prothrombin ratio	95.57 ± 3.74	95.13 ± 4.23	−0.44 ± 3.38	92.45 ± 5.69	92.29 ± 5.21	−0.16 ± 4.39
APTT	31.79 ± 3.57	32.11 ± 4.87	0.32 ± 2.39	31.70 ± 3.34	31.37 ± 3.16	−0.33 ± 2.51
fibrinogen	237.98 ± 38.83	240.22 ± 60.83	2.24 ± 63.04	231.21 ± 42.71	230.19 ± 40.38	−1.02 ± 53.94

## Data Availability

The data presented in this study are available on request from the corresponding author. The data are not publicly available due to ethical and privacy restrictions.

## Supplementary Materials

The following supporting information can be downloaded at: https://www.mdpi.com/article/10.3390/nu18040694/s1, Figure S1: Q–Q plots for log-transformed interleukin-6 values by group and time point. Note. Quantile-quantile plots for log-transformed interleukin-6 values. Points falling on or near the reference line indicate approximate normality. Minor deviations in the tails were observed but did not invalidate parametric analyses; Figure S2: Q–Q plots for log-transformed malondialdehyde values by group and time point. Note. Quantile-quantile plots for log-transformed malondialdehyde values. CG Time I shows deviation from normality due to floor effects; log-transformation improved distributional properties. Table S1: Summary of ANOVA results and effect sizes for primary inflammatory and oxidative stress outcomes.
