# Supplementary material for: Redox Water Consumption Attenuates Exercise-Induced Inflammation and Oxidative Stress in Physically Active Adults: A Randomized Controlled Trial
Source: Nutrients. 2026 Feb 21;18(4):694. doi: 10.3390/nu18040694 (PMC12943592; doi:10.3390/nu18040694)
Supplement: Supplementary file 1 [file nutrients-18-00694-s001.zip › nutrients-4103017-supplementary.pdf]

Two-Way Repeated Measures ANOVA (Log-IL-6)

The 2 × 2 repeated measures ANOVA on log-transformed IL-6 yielded a highly significant group × time interaction: $F(1,38) = 36.89$, $p < 0.001$, 95% CI [0.1568, 0.4837], large effect (Table 4). This indicates that the change in IL-6 from baseline to post-exercise differed substantially between the EG and CG. The main effect of time was also significant: $F(1, 38) = 6.23$, $p = 0.015$, 95% CI [0.005, 0.184], reflecting an overall shift in IL-6 across the sample. The main effect of group was not significant: $F(1, 38) = 0.11$, $p = 0.737$, 95% CI [0.0000, 0.0825], indicating comparable average IL-6 levels across groups when time is not considered (Table S1).

Two-Way Repeated Measures ANOVA (Log-MDA)

The 2 × 2 repeated measures ANOVA on log-transformed MDA yielded a significant group × time interaction: $F(1,38) = 4.98$, $p = 0.029$, 95% CI [0.0010, 0.1624] (medium effect). The main effect of time was significant: $F(1, 38) = 10.81$, $p = 0.002$, 95% CI [0.0351, 0.2359], and the main effect of group was also significant: $F(1, 38) = 6.22$, $p = 0.015$, 95% CI [0.0051, 0.1837]. These findings indicate differential temporal patterns of MDA between groups, with overall group differences in MDA levels (Table S1).

Table S1. Summary of ANOVA results and effect sizes for primary inflammatory and oxidative stress outcomes**.**

Outcome	Effect	F-value	p-value	R^2
Log(IL-6)	Group	0.1134	0.737214	0.3626
Log(IL-6)	Time	6.2269	0.014751	0.3626
Log(IL-6)	Group:Time	36.8942	0.0	0.3626
Log(MDA)	Group	6.2196	0.014808	0.2246
Log(MDA)	Time	10.8149	0.001527	0.2246
Log(MDA)	Group:Time	4.9794	0.028599	0.2246

Sensitivity and Model Diagnostics

Sensitivity analyses and model diagnostics confirmed the robustness of the parametric findings and are presented in Figure S1 (Supplementary Materials).

Residual plots from the primary ANOVA models showed acceptable distributions around zero with no systematic patterns. Q-Q plots indicated minor deviations from normality in the lower and upper tails, consistent with the Shapiro-Wilk test results but not sufficiently severe to invalidate the parametric analyses (Figure 4A and Figure 4B).

Non-parametric sensitivity analyses were conducted using Wilcoxon signed-rank tests (within-group comparisons) and Mann-Whitney U tests (between-group comparisons). For IL-6, non-parametric tests confirmed the parametric findings, with significant within-group changes in both EG

(Z = -3.47, p < 0.001) and CG (Z = 3.26, p < 0.001), and a highly significant between-group difference on change scores (Z = -4.12, p < 0.001). For MDA, non-parametric tests also validated the parametric results. Wilcoxon signed-rank tests showed no significant within-group change in the EG (Z = 0.5973, p = 0.5706), while the CG showed a non-significant trend toward increase (Z = 0.7840, p = 0.4524). Between-group comparison of MDA change scores using Mann-Whitney U test did not reach statistical significance (U = 151, Z = -1.3255, p = 0.1895), consistent with the parametric independent t-test (p = 0.241). These findings confirm robustness of conclusions across both parametric and non-parametric analytical approaches.

Analyses conducted on raw (non-transformed) IL-6 values yielded the same pattern of significance for the group × time interaction, confirming robustness of the findings. Exclusion of potential outliers (values > 3 SD from group mean) did not materially alter conclusions for any primary outcome (Figure S1)

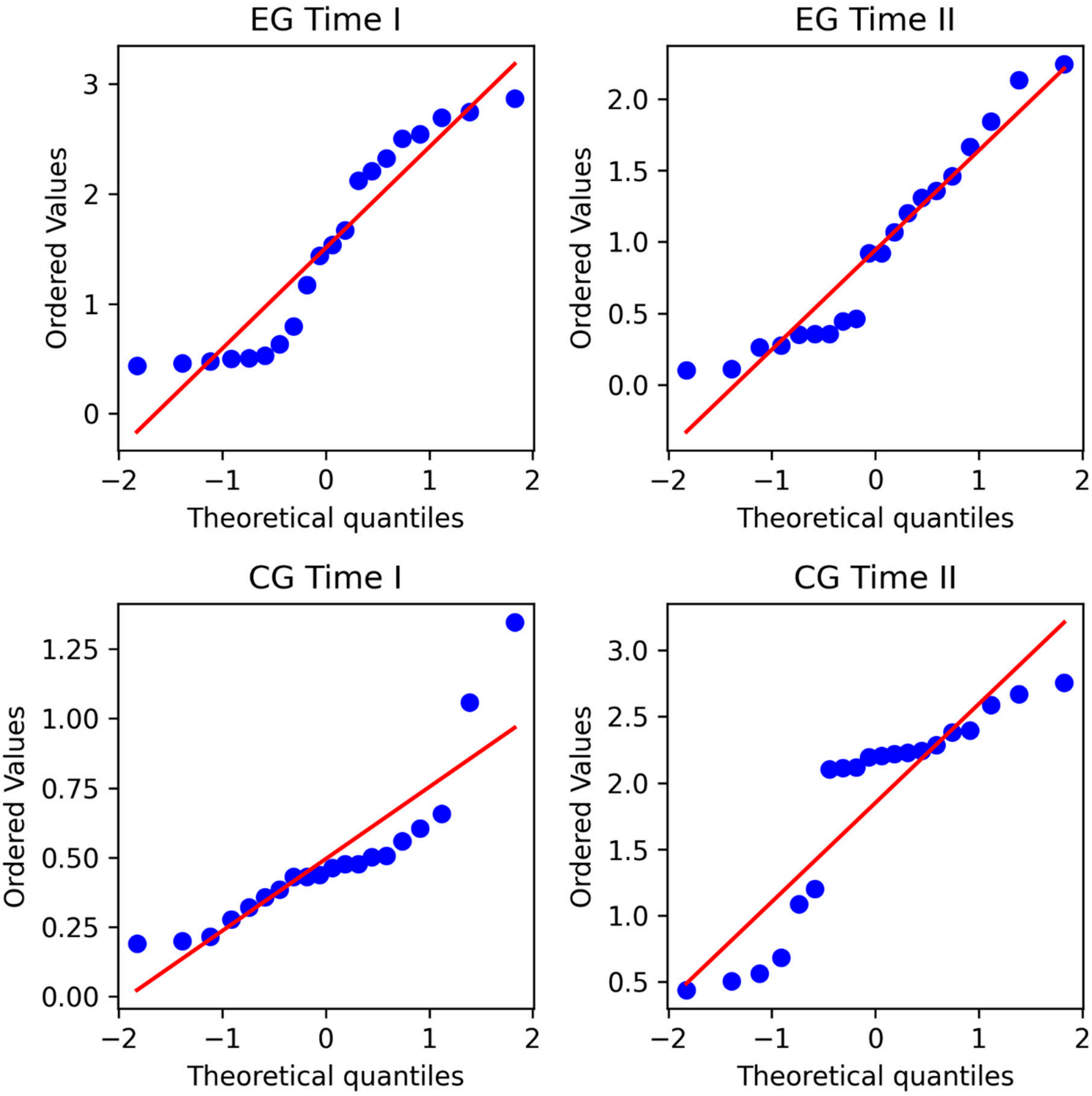

Figure S1. Q–Q plots for log-transformed interleukin-6 values by group and time point. Note. Quantile-quantile plots for log-transformed interleukin-6 values. Points falling on or near the reference line indicate approximate normality. Minor deviations in the tails were observed but did not invalidate parametric analyses.

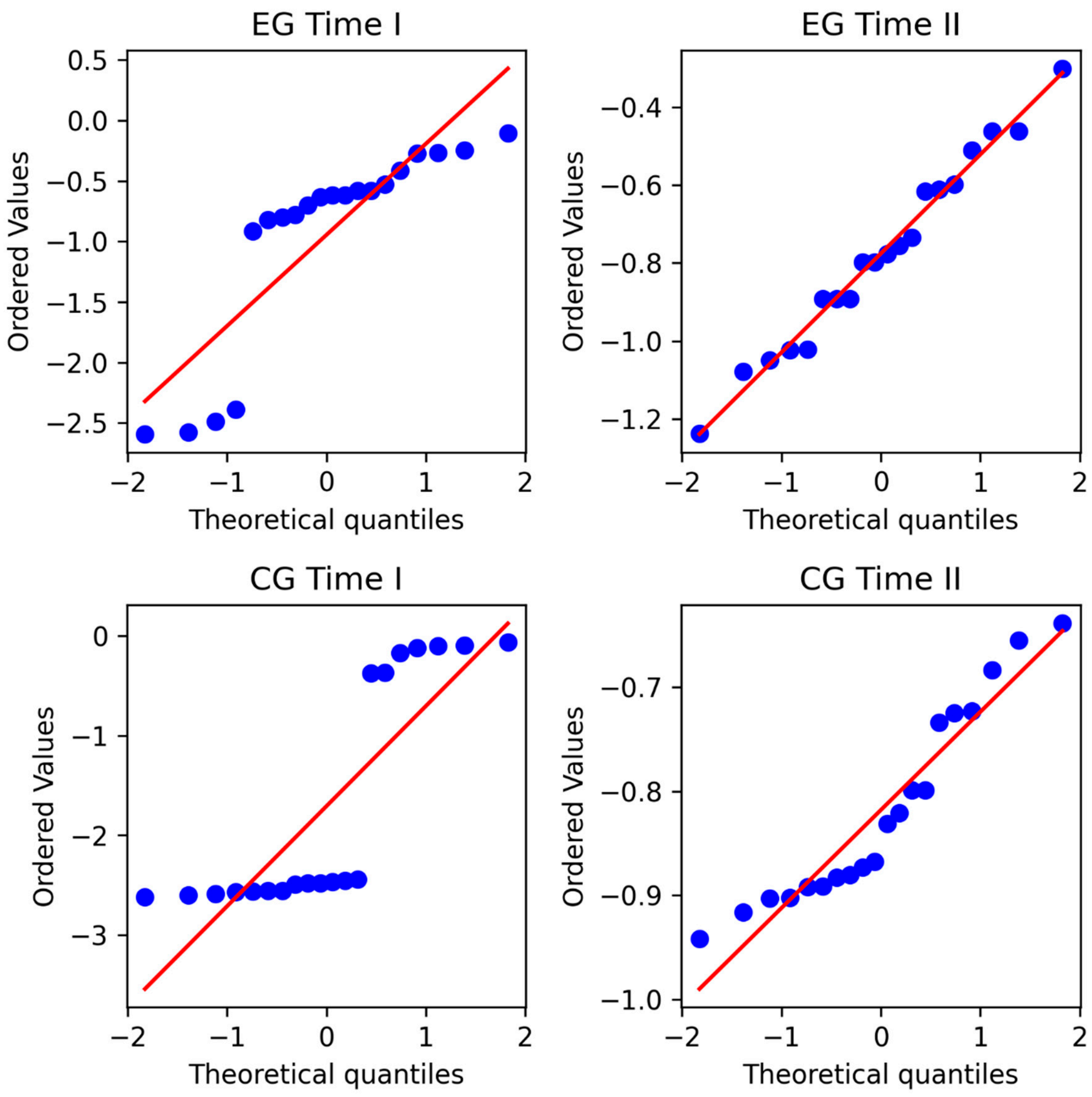

Figure S2. Q–Q plots for log-transformed malondialdehyde values by group and time point. Note. Quantile-quantile plots for log-transformed malondialdehyde values. CG Time I shows deviation from normality due to floor effects; log-transformation improved distributional properties.
